# Thiol-Amine Processed PbS Thin Films for Enhanced Near-Infrared Photodetection

**DOI:** 10.3390/nano16060363

**Published:** 2026-03-17

**Authors:** Yuanze Hong, Zhipeng Wei, Xiaohua Wang

**Affiliations:** State Key Laboratory of High Power Semiconductor Laser, College of Physics, Changchun University of Science and Technology, Changchun 130022, China; yzhong2023@sinano.ac.cn

**Keywords:** liquid-phase synthesis, PbS, film, photodetection, near-infrared

## Abstract

Developing reliable processing routes for semiconductor thin films is essential for advancing photodetection technologies. The amine-thiol solvent system, in comparison with other liquid-phase synthesis methods, does not necessitate stepwise ion-exchange reactions. It is capable of obtaining the target semiconductor thin film by directly dissolving bulk powder followed by subsequent annealing. Although PbO can be dissolved in this solvent as a raw material to obtain PbS thin films, the structural evolution, optical properties, and photodetection performance of the films obtained via this solvent system still require further exploration. This solvent system was employed to prepare PbS thin films, and a comprehensive investigation was carried out on the evolution of their structure, morphology, and optical properties during preheating and annealing treatments. During preheating, the films exhibit directional ordering within the organic matrix, which converts into phase-pure PbS upon annealing. Based on the optimized films, interdigitated photodetectors and hybrid devices integrated with graphene transistors are fabricated. The resulting devices exhibit strong photoresponse and operational stability, demonstrating the viability of amine-thiol-processed PbS films for photodetection applications.

## 1. Introduction

Infrared band detection serves as a fundamental cornerstone of contemporary sensing and imaging technology, and it is extensively utilized in various fields such as military night vision, industrial thermal imaging, medical diagnosis, and environmental gas monitoring. Bulk thin films of PbS, characterized by a narrow band gap of approximately 0.41 eV and a high light absorption coefficient, demonstrate outstanding room-temperature photoresponse performance within the 1–3 μm near-infrared band [1,2,3,4,5]. Currently, the primary methods for preparing PbS thin films are magnetron sputtering, atomic layer deposition and thermal evaporation [5,6,7]. All of these preparation methods depend on large-scale equipment and high-vacuum environments, which undoubtedly elevate the preparation cost and initial investment. Although the chemical bath deposition (CBD) method enables low-cost preparation using simple equipment, a multitude of experimental parameters, including the pH of the solution, the type and concentration of the precursor, and the growth temperature, all influence the final film morphology. This influence results in the complexity of replicating experimental results [8,9,10]. Meanwhile, as the reaction progresses, the concentration of the reactants will decline, which renders the control of the film-formation process challenging and also restricts the final thickness of the film [11,12]. Therefore, a more advanced manufacturing method is yet to be developed, which should be characterized by simple synthesis, easy processing, and the ability to restore highly crystalline PbS films under mild conditions.

In 2013, Brutchey reported a binary solvent composed of ethylenediamine (EN) and ethylenethiol (EDT), which can dissolve nine kinds of metal sulfides and is called alkahest [8]. Unlike the step-by-step ion-exchange method employed in the CBD approach for the preparation of semiconductor films, this solvent system is capable of directly dissolving the target semiconductor powder to yield semiconductor ink at the molecular level. Via mild annealing, spin-coated semiconductor ink deposited on the substrate is converted into high-quality semiconductor films [9,10,11]. As the dissolution capacity of this solvent combination is continuously investigated, the system of this solvent has also been expanded, and various solvent combinations have emerged, such as EN-mercaptoethanol (ME) [12], propylamine-EDT [13], and ethanolamine-thioglycol [14,15]. The substances that can be dissolved have expanded from the initial metal sulfides to metal oxides, metals, and non-metallic elements [16]. A comprehensive insight into and understanding of the form of solute are essential for controlling and optimizing the formation of semiconductor films. Through mass spectrometry analysis, it was determined that the Sb_2_S_3_ dissolved in EN and ME could be classified into two types of components in the solution: one type contains one Sb atom, specifically [SbS][C_2_H_4_SO][C_2_H_9_N_2_] and [Sb][C_4_H_8_S_2_O_2_][C_2_H_9_N_2_]; the other type contains two Sb atoms, specifically [Sb_2_S_3_][C_2_H_4_SO][C_2_H_9_N_2_] and [Sb_2_S_2_][C_4_H_9_S_2_O_2_][C_2_H_9_N_2_]. The existence forms of these complexes were also verified by FTIR spectra [17]. To explore the molecular products of superhydride-treated Sb_2_Te_3_ dissolved in EN and EDT, Seungki carried out a component analysis on the precipitate subsequent to the addition of acetonitrile. The analysis revealed that the atomic ratio of the molecular solute was 2:7 (Sb:Te), which was in accordance with the dimer cluster Sb_2_Te_7_^4-^ synthesized in hydrazine [18]. By continuously extracting excess Te from the solution using TOP, a molecularly homogeneous precursor ink was obtained. Subsequent processing of this ink enabled the fabrication of high-quality and dense films. However, if the solution is not treated with superhydride or the excess Te is not removed, the quality of the formed film will be significantly inferior. These working descriptions suggest that within the precursor solution, the solute might exist either in a form coordinated with thiol or in a cluster form, whereas the solvent functions as an anti-ion to uphold charge balance [19]. Currently, the films obtained via this solvent system have been successfully applied in the field of photodetection [12,14,20]. In the earlier research, an attempt was made to prepare SnS as a thin film using this solvent combination, and the pure-phase SnS could be obtained at 350 °C. The photoresponse of SnS on FTO substrates was examined by means of electrochemical methods. A photocurrent of 170 μA cm^−2^ was achieved, which holds a significant advantage over the films prepared by electrodeposition (6 μA cm^−2^) [21]. Wang achieved a pure-phase PbSe thin film with a band gap of 0.26 eV by annealing the ink containing Pb, diphenyl diselenide, and EN at 400 °C [22]. After the sensitization annealing, the photoconductive PbSe detector attained the maximum photoresponse value (6.97 A/W) and an external quantum efficiency (EQE) of 247% at a wavelength of 3.5 μm under room temperature conditions [23]. The distinctive morphology and the composite composition resulting from annealing could potentially be the factors contributing to the high gain of this device. The preparation method of PbS thin films has also been developed [10]. PbO, serving as the lead source, is dissolved in a mixed solvent of EN and ME, and pure-phase PbS thin films can be obtained via mild annealing. However, to date, the surface morphology, optical properties, and suitability of PbS films prepared by this method for photodetection remain unclear.

In this work, PbS thin films were synthesized using EN and ME solvent systems. The alterations in the structural, morphological, and optical properties of this molecular ink were examined via preheating at 110 °C and annealing at 350 °C. It was discovered that during the pre-heating stage, the film contained an intermediate phase, which underwent a transformation into the pure-phase PbS at 350 °C. Ultimately, the feasibility of its photodetection was verified via the interdigital electrode device. Moreover, it was also confirmed through hybrid devices composed of PbS thin films and graphene transistors that such PbS thin films can satisfy the requirements of different types of photodetectors.

## 2. Materials and Methods

### 2.1. Materials

Ethylenediamine was purchased from Sinopharm Chemical Reagent Co., Ltd. (Shanghai, China). 1,2-Ethanedithiol and 2-Mercaptoethanol were purchased from Shanghai Macklin Biochemical Co., Ltd. (Shanghai, China). PbO was purchased from Aladdin Reagent (Shanghai) Co., Ltd. (Shanghai, China). All reagents and solvents were used as received without further purification.

### 2.2. Preparation of PbS Film

The preparation of the PbS precursor solution was carried out in reference to a previous report [10]. A total of 200 mg of PbO was dissolved in a mixed solution consisting of 1 mL of ethylenediamine and 0.25 mL of 2-mercaptoethanol. The solution was magnetically stirred at room temperature for 30 min to yield a transparent yellow solution. To investigate the potential changes occurring during the transition of the PbS film from the precursor solution to the final crystallization, two temperatures were set: 110 °C for pre-heating to evaporate the solvent and 350 °C for high-temperature annealing to initiate crystallization. The preparation schematic diagram is shown in Figure S1.

### 2.3. Device Fabrication

The chemical vapor deposition (CVD)-grown monolayer graphene film on Si/SiO_2_ (500 nm) substrate was purchased from Nanjing XFNANO Materials Tech Co., Ltd. (Nanjing, China). Using the MA6 system, UV lithography and plasma etching are employed to pattern the graphene channel, followed by electron beam evaporation for depositing Cr/Au metal electrodes with thicknesses of 15 nm and 65 nm, respectively. The graphene channel length (L) and channel width (W) were defined as 5 and 115 μm, respectively. The preparation of the hybrid device was accomplished by depositing the precursor solution of PbS onto the graphene transistor, baking it at 110 °C for 20 min, and subsequently annealing it at 350 °C for 30 min. The interdigitated electrodes are obtained by evaporating Cr/Au metal electrodes with thicknesses of 15 nm/65 nm on the Si/SiO_2_ wafer through a mask. The model of the electron beam evaporation equipment is Ebe-07 (deposition rate: 0.1 nm/min). The interdigitated electrode was initially subjected to a 1 min hydrophilic treatment in an oxygen plasma (80 W). The PbS precursor ink was spin-coated onto the interdigitated electrodes for the fabrication of the device. Subsequently, the substrate was baked at 110 °C for 1 min, and the spin-coating and heating process was repeated five times.

### 2.4. Characterization

Powder X-ray diffraction (XRD) patterns were recorded on a D8 Advance powder X-ray diffractometer (Bruker, Shanghai, China), using Cu-Kα radiation (λ = 1.54056 Å). Thermogravimetric analysis (TGA) was performed on a TA Instruments TG209F1 instrument (Netzsch, Shanghai, China) with an alumina crucible under a flowing nitrogen atmosphere with a heating rate of 10 °C/min. Fourier transform infrared spectroscopy (FTIR) experiments were performed on the equipment of Nicolet 6700 (Bruker, Beijing, China). The absorption spectra were measured with an ultraviolet-visible-near-infrared spectrophotometer (GENESIS 150, Thermo Fisher Scientific, Shenzhen, China). Surface morphology was characterized by a scanning electron microscope (SEM) using a Nova NanoSEM450 (Thermo Fisher Scientific, Shanghai, China).

## 3. Results and Discussion

The dissolution capacity of the dual-solvent system developed by Brutchey has increased from the 9 metal sulfides reported in 2013 to more than 100 bulk materials as of now, encompassing metal sulfides, metal oxides, metals, and non-metallic elements [16,21,24]. The advantage of this solvent combination compared to other solution synthesis methods lies in the fact that it does not necessitate precise control of metal salts and strict reaction conditions to govern the reaction. Instead, it directly synthesizes the precursor ink from the target compound or the elemental composition of the target compound [10,25]. The ink applied on the substrate can be transformed into a crystalline semiconductor film through gentle annealing [15,26]. This straightforward synthesis and processing approach cannot be replicated by other synthesis methods.

**Figure 1:**
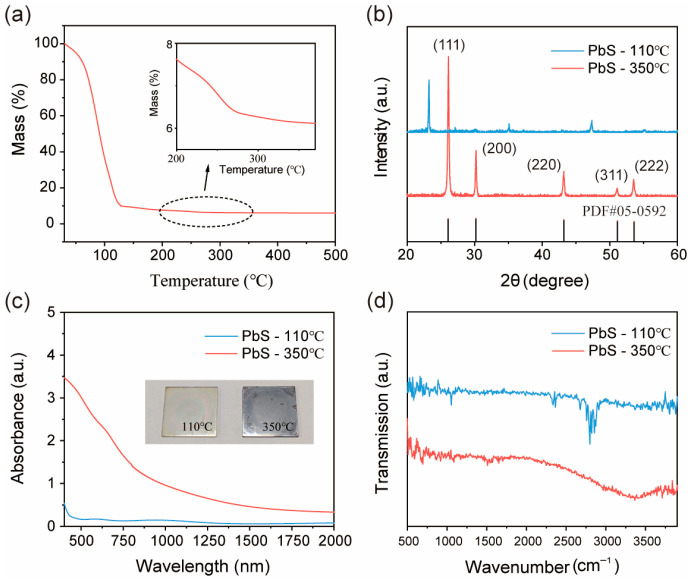
(**a**) thermogravimetric analysis of the PbS precursors prepared in EN and ME. (**b**) X-ray diffraction patterns of products recovered from PbS precursors after pre-heating at 110 °C and annealing at 350 °C. (**c**) UV–vis absorption spectroscopy for PbS thin films after pre-heating at 110 °C and annealing at 350 °C. (**d**) Fourier transform infrared spectroscopy for PbS thin films after pre-heating at 110 °C and annealing at 350 °C.

Firstly, TGA was performed in Figure 1a on the PbS precursor solution to determine the temperature at which it could be converted into a semiconductor film in the subsequent process. A substantial reduction in mass takes place at approximately 120 °C, which can be attributed to the evaporation of the primary solvent (EN boiling point: 116 °C). Subsequently, the curve gradually declines to approximately 260 °C. During this process, the organic substances in the system continue to volatilize and gradually reach the crystallization temperature [21]. After reaching 300 °C, the mass curve tends to stabilize; therefore, 350 °C is selected as the annealing temperature.

The structural properties of the PbS thin film were determined via XRD, as depicted in Figure 1b. After undergoing annealing at 350 °C, the diffraction peaks of the sample were detected at 26.1°, 30.2°, 43.2°, 51.1°, and 53.5°, corresponding to the (111), (200), (220), (311), and (222) crystal planes of PbS (PDF#05-0592), respectively [10,27]. The average crystalline size (*D*) was calculated using Debye-Scherrer’s formula [28]:

(1) $$D=\frac{K\lambda }{\beta \cos\theta }$$

where *K* is Scherer’s constant and is equal to ~0.9, *λ* is the X-ray wavelength of CuKα radiation and equals 0.154 nm, *θ* is the Bragg diffraction angle, and *β* is the FWHM of the XRD peak appearing at the diffraction angle *θ*. The strongest (111) peak was used for calculation, and the average crystal size was 40.7 nm. The lattice constant was calculated by Jade 6.5 software as 0.59452 nm, which is consistent with the reported value [29]. Interestingly, a distinct peak position at 23.2° was detected in the PbS thin film subsequent to pre-heating at 110 °C. However, these peak positions cannot be found to correspond to Pb or S species in the PDF card, which might be attributed to an oriented arrangement involving organic components [24]. Indeed, when metals dissolve in this binary solvent, thiol groups coordinate with metal ions to form complexes, whereas small amine molecules serve as counterions to maintain charge balance [17,24,30]. For example, in the reaction of synthesizing PbSe from EN and diphenyl diselenide, diphenyl diselenide coordinates with Pb ions to produce Pb(SePh)_2_ as the crystalline product [24]. Based on these reports, it can be deduced that in the solvents of EN and ME, Pb ions coordinate with ME, while EN functions as an anti-ion. After being heated at 110 °C, Pb(C_2_H_4_SO)_2_ in the film has undergone directional arrangement [24]. According to TGA, subsequent to annealing at 350 °C, the organic matter was removed, and the crystalline state of the film reverted to pure-phase PbS.

The optical absorption spectra of the two films are presented in Figure 1c. For the pre-heated samples, there was nearly no absorption beyond 500 nm, which also corresponded to the nearly transparent quartz plate samples. The absorption prior to 500 nm can be ascribed to the complex molecules formed by Pb ions in the solution and the solvent [31,32]. After annealing, the sample exhibited a silver-gray color, and the absorption intensity of the sample increased across the entire spectral range. This characteristic is beneficial for the sample’s application as a photosensitive material. The optical band gap of the annealed PbS thin film was estimated to be 1.51 eV through the application of the Tauc model [3], as illustrated in Figure S2. The alterations in the organic components of the film at various stages were characterized by FTIR, as depicted in Figure 1d. The absorption within the range of 2700 cm^−1^ to 2900 cm^−1^ can be ascribed to the stretching vibrations of C-H and N-H bonds in the solvent system [21,33]. The presence of the 1052 cm^−1^ band can be associated with the C-O vibration originating from ME [17]. The complete disappearance of the signals of these organic substances in the annealed sample suggests the purity of the sample, which is also consistent with the XRD results.

**Figure 2:**
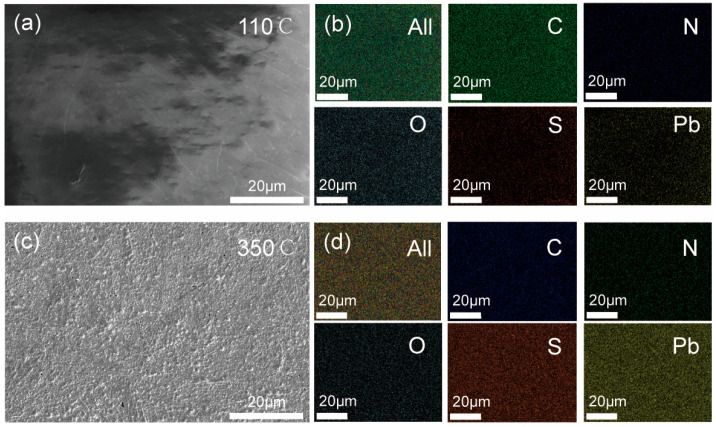
(**a**) SEM image and (**b**) mapping of PbS thin film preheated at 110 °C. (**c**) SEM image and (**d**) mapping of PbS thin film annealed at 350 °C.

Figure 2 depicts the morphology and elemental composition of the PbS thin film. As depicted in Figure 2a, the PbS thin film pre-heated at 110 °C exhibits a smooth surface. Element mapping indicates that the five elements, namely C, N, O, S, and Pb, are uniformly distributed within the film, and their atomic number ratios are 45%, 6%, 29%, 10%, and 10%, respectively. After annealing at 350 °C (Figure 2c), the surface of the PbS thin film consists of aggregated nanoparticles and features a few pores, which could potentially be attributed to the volatilization of organic substances. The morphology of the film under high-magnification observation is presented in Figure S3, which clearly shows that the film consists of aggregated nanoparticles. The elemental distribution indicates that the annealing process at 350 °C still preserves a uniform distribution of elements; however, their proportions have undergone changes: C (11%), N (4%), O (10%), S (35%), and Pb (40%). This indicates that a substantial quantity of organic components has escaped during the high-temperature annealing process, which is in accordance with the results of FTIR. To demonstrate the advantages of the solvent system in processing films, films of different thicknesses were obtained by adjusting the number of spin-coating times, as shown in Figure S4. When the number of spin-coating cycles was increased from 2 to 8, the thickness of the PbS film increased from 217 to 381 nm, with an average increase of approximately 27 nm per spin-coating cycle. This suggests that the solvent combination can be employed to control the film thickness through spin-coating.

**Figure 3:**
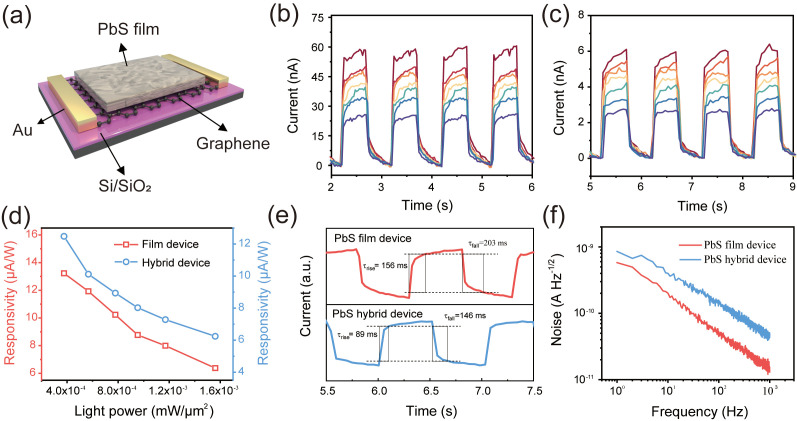
(**a**) Schematic of the hybrid device model. The photocurrent performance of the PbS film device (**b**) and hybrid device (**c**) under varying light power densities (808 nm). (**d**) The responsivity of the two devices under 808 nm excitation at different power densities. (**e**) The response times of the two devices. (**f**) Dark current noise density as a function of frequency.

Two types of devices were fabricated to verify the photoelectric detection capability of the PbS thin film: one was a thin-film device with interdigitated electrodes, and the other was a hybrid device integrated with a graphene transistor. For the interdigitated electrode thin-film devices, the electrodes were initially treated with oxygen plasma for 1 min to render their surfaces hydrophilic. To guarantee that the films had adequate thickness, the spin-coating and heating process was replicated five times. The final device is presented in Figure S5. The device was excited using an 808 nm laser, and the variations in photocurrent were recorded, as depicted in Figure 3b. Even with such a straightforward device structure, the photoresponse of the PbS thin film remains highly sensitive and stable. When the light power density is at its maximum (1.56 × 10^−3^ mW/μm^2^), the net photocurrent of the device is 49 nA. As the optical power density decreases to 3.76 × 10^−4^ mW/μm^2^, the net photocurrent of the device declines to 25 nA. To verify the uniformity of the PbS films prepared by this method, four interdigital devices were tested. All of these devices exhibited stable photoresponse, as depicted in Figure S6. The similar photocurrent magnitudes prove the uniformity and reproducibility of the PbS film. The photographs of the hybrid device prior to and subsequent to spin coating are presented in Figure S7. The photograph after annealing indicates that the graphene channel is completely covered with PbS. This device exhibits a discernible response to 808 nm lasers of varying powers, as depicted in Figure 3c. Although the photocurrent is merely a few nanoamperes, the fluctuating state of the response current remains clearly distinguishable. The responsivity of the two devices is presented in Figure 3d. The responsivity (808 nm) of the thin-film device and the hybrid device was 13.2 and 12.5 μA/W, respectively, when excited at a power density of 3.76 × 10^−4^ mW/μm^2^. The response time of the device is presented in Figure 3e. The rise time and fall time of the thin-film device are 156 and 203 ms, respectively. In the hybrid devices, the response time was shortened to 89 and 145 ms, respectively, which indicates that the introduction of graphene is more conducive to the collection of photogenerated carriers. The dark current noise density of both devices is on the order of 10^−10^ A Hz^−1/2^. The specific detectivity of the device is shown in Figure S8. The specific detectivities of the thin-film device and the hybrid device are 1.3 × 10^11^ and 8.9 × 10^10^ Jones, respectively. The parameters of the two devices are shown in Table S1. Finally, the stability of the hybrid device was also investigated. As depicted in Figure S9, no decline in photocurrent was detected over approximately one hundred cycles. The performance of the device remained stable after one-week storage, which demonstrated the stability of both the thin film and the device.

## 4. Conclusions

Pure-phase PbS thin films were successfully prepared by using PbO in a mixed solvent of EN and ME. This film exhibits an intermediate phase at 110 °C, which will be completely transformed into PbS during the annealing process at 350 °C. This film consists of nanoparticles and contains a small number of voids. Thin-film devices consisting of interdigital electrodes and hybrid devices composed of graphene transistors are employed to detect photoelectric performance. In both types of devices, a clear and stable photoresponse was observed, thereby demonstrating that the PbS thin films prepared by this method can be applied in the field of photodetection.

## Data Availability

Data is contained within the article or Supplementary Material.

## Supplementary Materials

The following supporting information can be downloaded at: https://www.mdpi.com/article/10.3390/nano16060363/s1, Figure S1: The schematic diagram; Figure S2: (αhν)^2^ as a function of hν for of the annealed PbS Films; Figure S3: SEM image of PbS thin film annealed at 350 °C. Figure S4: The number of spin-coating cycles and the corresponding thickness of PbS thin films; Figure S5: Digital photos of the interdigitated electrode device; Figure S6: The photoresponse performance (a) and photocurrent statistics (b) of four interdigitated electrode devices; Figure S7: Photos of the hybrid device before spin coating (a) and after annealing (b). Figure S8: The specific detectivity of the two devices under 808 nm excitation of different power densities. Figure S9: The photoresponse stability test was conducted on the newly prepared samples (a) and those after one week (b); Table S1: Parameters of PbS photodetectors.
