# Supplementary material for: Thiol-Amine Processed PbS Thin Films for Enhanced Near-Infrared Photodetection
Source: Nanomaterials (Basel). 2026 Mar 17;16(6):363. doi: 10.3390/nano16060363 (PMC13029462; doi:10.3390/nano16060363)
Supplement: Supplementary file 1 [file nanomaterials-16-00363-s001.zip › nanomaterials-4182193-supplementary.pdf]

Thiol-amine Processed PbS Thin Films for Enhanced Near-infrared Photodetection

Yuanze Hong, Zhipeng Wei * and Xiaohua Wang *

State Key Laboratory of High Power Semiconductor Laser, College of Physics, Changchun University of Science and Technology, Changchun 130022, China; yzhong2023@sinano.ac.cn

* Correspondence: zpweicust@126.com (Z.W.); biewang2001@126.com (X.W.)

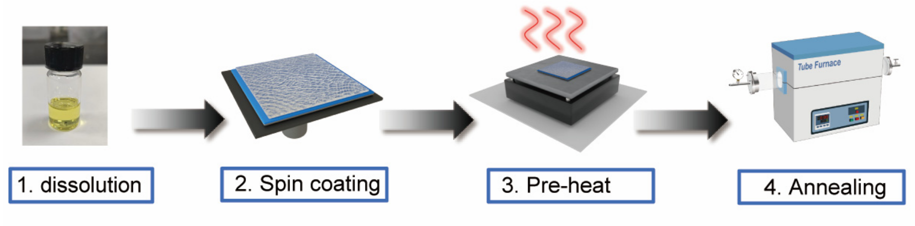

Figure S1. The schematic diagram depicting the transformation from the PbS precursor solution to the final crystalline film encompasses spin coating, preheating, and annealing processes, respectively.

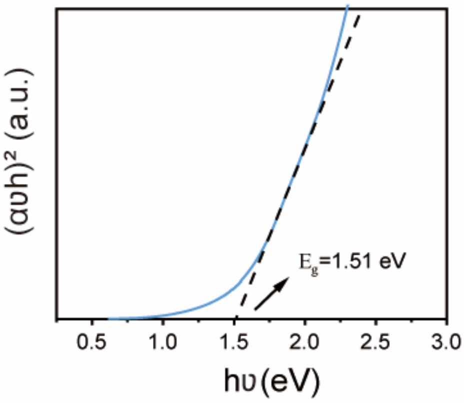

Figure S2. $(\alpha h\nu)^2$ as a function of hν for of the annealed PbS Films.

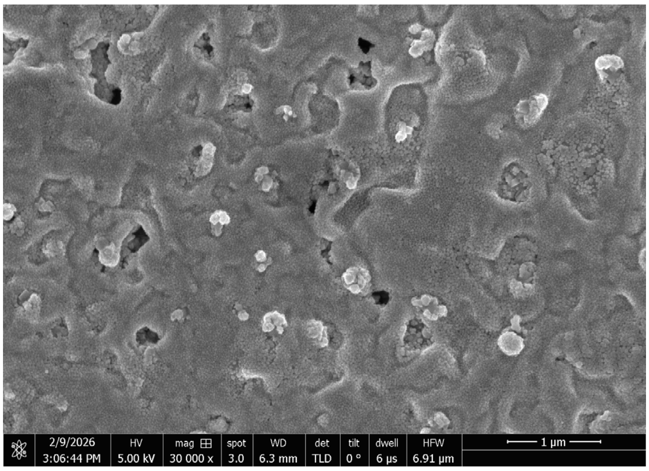

Figure S3. SEM image of PbS thin film annealed at 350°C.

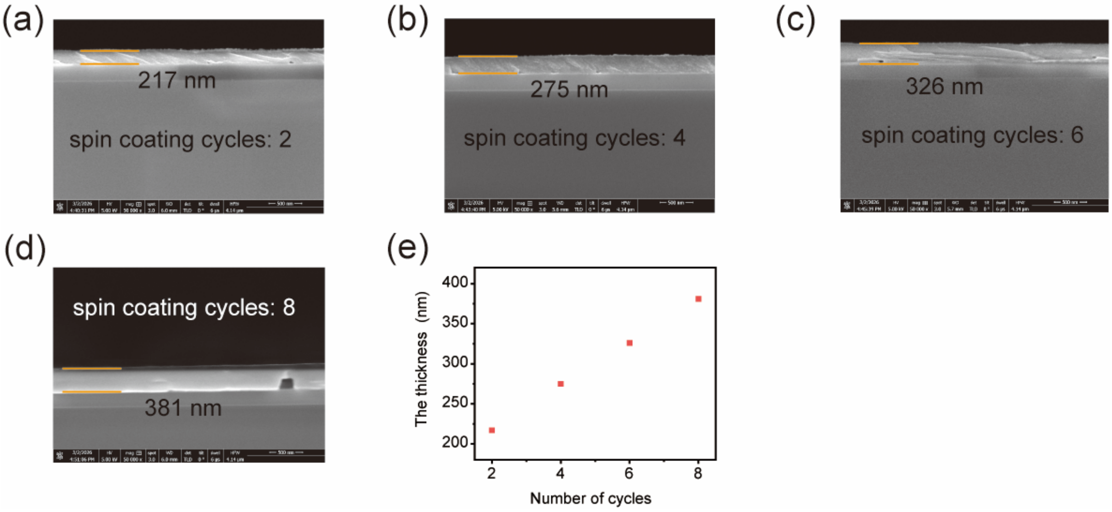

Figure S4. The number of spin-coating cycles and the corresponding thickness of PbS thin films.

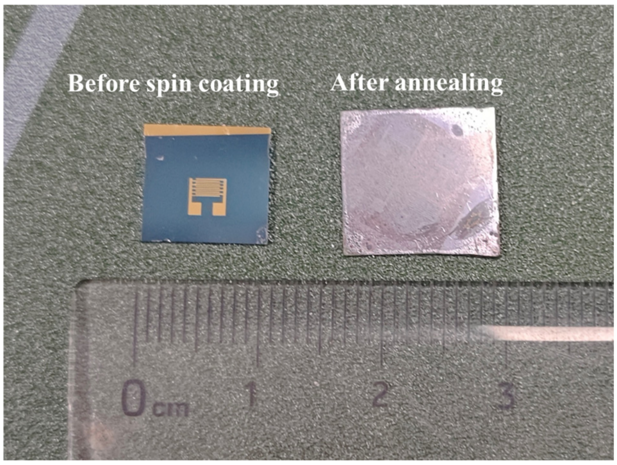

Figure S5. Digital photos of the interdigitated electrode device.

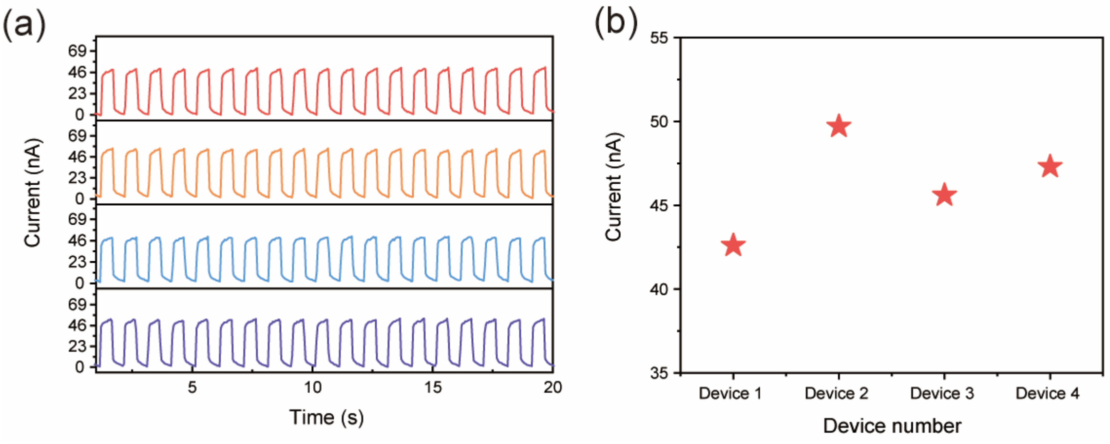

Figure S6. The photoresponse performance (a) and photocurrent statistics (b) of four interdigitated electrode devices

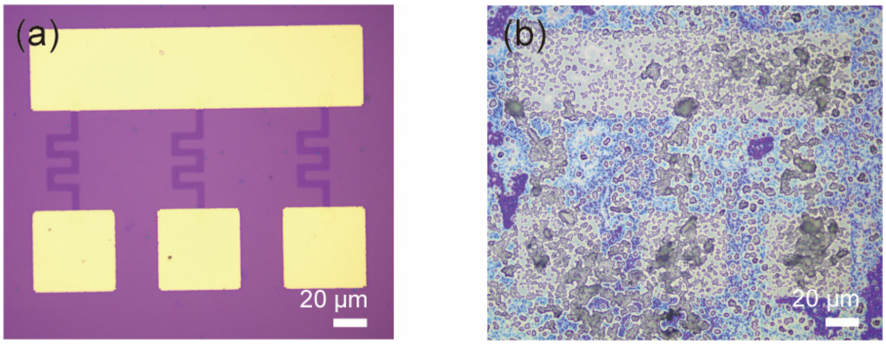

Figure S7. Photos of the hybrid device before spin coating (a) and after annealing (b).

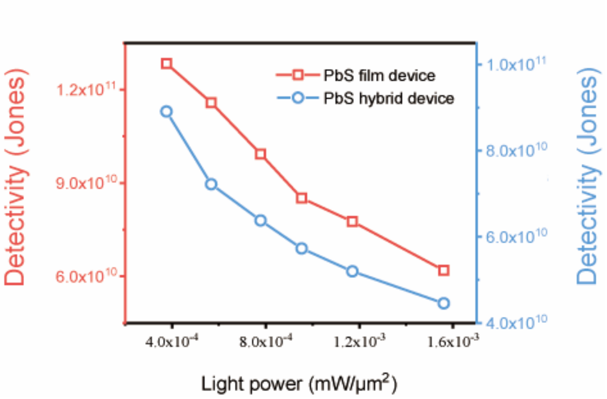

Figure S8. The specific detectivity of the two devices under 808 nm excitation of different power densities.

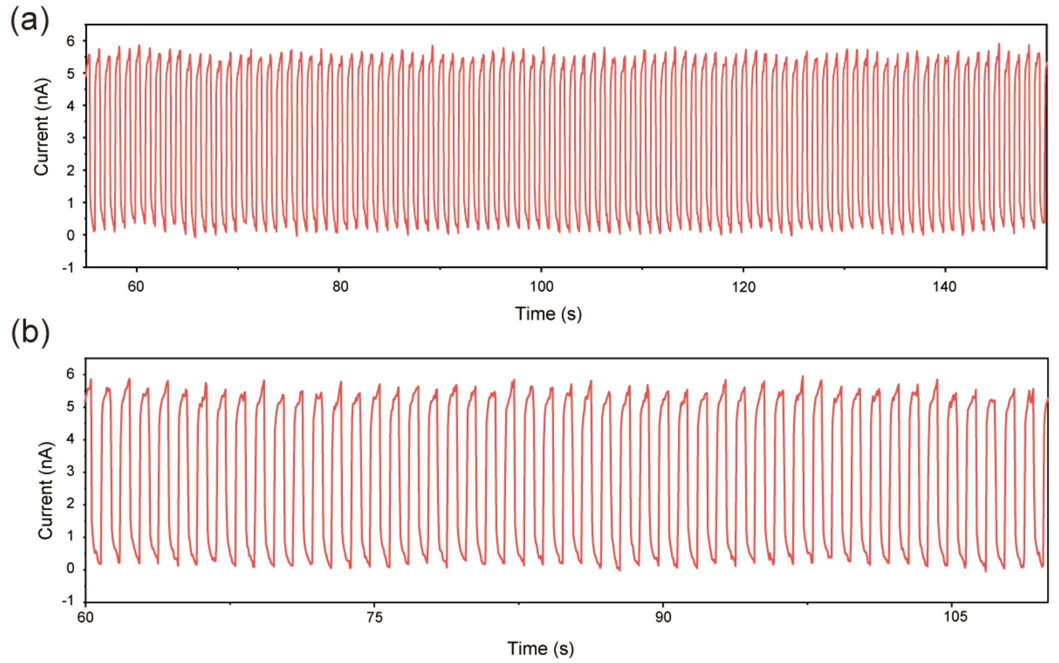

Figure S9. The photoresponse stability test was conducted on the newly prepared samples (a) and those after one week (b).

Table S1. Parameters of PbS photodetectors

Device type	R (μA/W)	Noise (A $Hz^{-1/2}$)	D* (Jones)
Thin-film devices	13.2	5.79×10^{-10}	1.3×10^{11}
Hybrid devices	12.5	8.54×10^{-10}	8.9×10^{10}
